# Fracture behaviour assessment of high-performance fibre-reinforced concrete at high strain rates using interpretable modelling approaches

**DOI:** 10.1016/j.heliyon.2024.e24704

**Published:** 2024-01-17

**Authors:** Quang Dang Nguyen, Khoa Tan Nguyen, Tuan Kiet Tran, Kihak Lee, An Thao Huynh

**Affiliations:** a Centre for Complex Systems, Faculty of Engineering, The University of Sydney, New South Wales, Australia; b Institute of Research and Development, Duy Tan University, 550000, Da Nang, Viet Nam; c Department of Civil Engineering, Ho Chi Minh City University of Technology and Education, Ho Chi Minh, 700000, Viet Nam; d Deep Learning Architectural Research Center, Department of Architectural Engineering, Sejong University, Seoul, 05006, South Korea; e School of Built Environment, Engineering and Computing, Leeds Beckett University, City Campus, Leeds, LS1 3HE, UK

**Keywords:** High-performance fibre-reinforced concrete, Machine-learning-based modelling, Global sensitivity analysis, Fracture strength analysis, Interpretable approach, Proactive failure analysis

## Abstract

High-performance fibre-reinforced concrete (HPFRC), a type of cementitious composite material known for its exceptional mechanical performance, has widespread applications in structures exposed to severe dynamic loading conditions. However, understanding nonlinear HPFRC fracture behaviour, particularly under high strain rates, remains challenging given the complexities of assessment procedures and cost-intensive nature of experiments. This study presents an interpretable framework for modelling and analysing HPFRC fracture strength at high strain rates. A wide range of machine learning methods, including ensemble techniques, were employed to capture multivariate effects of eight essential input features (e.g., mortar compressive strength, fibre physical and mechanical properties, cross-sectional area, and strain rate) on fracture strength response. To assess the derived models, a novel evaluation procedure was proposed involving a data-based analysis, employing established metrics (i.e., coefficient of determination, root mean squared error, and mean absolute error via K-fold cross-validation) and a domain experts-involved evaluation utilising global sensitivity analysis to discern first-order and higher-order interactions among input factors. The proposed approach efficiently yielded both quantitative and qualitative insights into crucial input factors governing HPFRC fracture strength with limited experimental data. The obtained findings highlight the significance of multivariate effects, such as the interaction between strain rate and fibre tensile strength, and between fibre volume and fibre diameter, on fracture behaviour. The proposed interpretable framework aims to provide a powerful tool for proactive material failure analysis by understanding fracture behaviour and identifying potential weak and strong interactions among input factors of HPFRC-based samples. Moreover, the utilisation of the proposed approach enables researchers and civil engineers to efficiently focus on the most critical input parameters during the early design stage and ensuring the structural integrity and safety of HPFRC-based constructions.

## 1 Introduction

High-performance fibre-reinforced concrete (HPFRC) has become a widely used material in the construction industry due to its exceptional energy absorption capacity [1]. This material incorporates natural or synthetic fibres into the cementitious matrix of concrete to enhance its mechanical properties. Steel fibres, in the form of straight, twisted, or hooked shapes, are often integrated into HPFRC to bridge cracks and prevent crack growth, compensating for typical issues associated with low tensile strength and resistance to impact forces in plain concrete structures. HPFRC containing less than 2.5 % by volume of randomly oriented steel fibres was reported to possess high compressive strength within the range of 160–250 MPa and tensile strength exceeding 8 MPa [[2], [3], [4], [5], [6], [7]]. Therefore, its outstanding durability enables a reduction in size of structural elements, of up to 30 % by weight compared to conventional reinforced concrete structures [8]. HPFRC has been used in various applications including beams [3] and façade panels [9] which require high impact resistance, particularly to mitigate the adverse consequences of extreme events such as typhoons, earthquakes, tsunamis, and explosions [3,4].

Fracture strength describing the fracture behaviours of HPFRC structures is considered as a quantitative parameter to analyse their brittleness and cracking resistance. Previous studies [10,11] indicate that fracture strength primarily depends on physical and mechanical properties of fibres, cementitious matrix strength and strain rate. HPFRC specimens exhibit tensile-hardening behaviour at high strain rates ranging from 5 to 92 s^−1^, with improved strength due to the rate-sensitive interfacial bond characteristics between the cementitious matrix and fibres [12]. To measure HPFRC fracture strength at high strain rates, Tran and Kim [13] proposed the use of a strain energy frame impact machine (SEFIM). This testing system, comprising a high-speed camera and dynamic strain gauges, was developed to analyse direct tensile behaviour of HPFRC beams at high strain rates of up to 92 s^−1^. Another testing system to evaluate HPFRC fracture strength is the fibre optics brag grating sensor introduced by Wahba and Marzouk [14], where fibre optic strain gauges were attached to a testing machine to determine the stress-strain relationship of large-sized HPFRC beams with length of up to 1 m. Uniaxial tensile tests were also conducted on double-bell-shaped HPFRC specimens to examine their tensile behaviour [[15], [16], [17]].

While laboratory experiments are effective in providing direct insights into HPFRC fracture behaviour under high-rate loading, significant challenges remain due to limitations in standardising testing criteria, expensive equipment and materials and other issues related to machine frame stability, gripping and eccentricities [18]. Dang and Kim [19] assessed the effect of strain rate, fibre volume and types on fracture behaviour of ultra-high-performance fibre-reinforced concrete (UHPFRC) incorporating nanoparticles under high strain rate ranging from 0.000333 to 156.55 s^−1^. The testing UHPFRC beams showed high rate-sensitive fracture resistance when strain rate increased. This experimental study focused solely on the impact of individual input features without considering the combined effects of multiple input features. Multivariate effects of various factors such as fibre content, mortar compressive strength and curing age on UHPFRC properties were investigated in the study by Zou et al. [20]. However, this study primarily focused on UHPFRC compressive strength at static loading conditions. Designing UHPFRC structures to withstand dynamic loading conditions requires a comprehensive understanding of their fracture behaviour under high strain rate. This highlights the need for an efficient approach to evaluate effects of multiple input features on HPFRC fracture strength.

Despite extensive research on quasi-static behaviour of HPFRC structures [[21], [22], [23]], findings on fracture properties of such material under high-rate loading is limited due to the complexity of experiments involved. A thorough literature search revealed that there has been no comprehensive research on the use of data-driven approaches to analyse HPFRC fracture behaviour subjected to high strain rates. One of the major barriers to this adoption is the scarcity of data available to facilitate this investigation. Specifically, evaluating the performance of machine learning (ML) models with small sample sizes poses a significant challenge. Randomly selecting training and validation data [22,23] may not eliminate data biasing and randomness in assessing prediction models. Other techniques, such as K-fold cross-validation, were employed to eliminate randomness from performance evaluation [5,[24], [25], [26]], but they were still limited by small-size data constraints that prevent them from overcoming the potential problem of data biasing and overfitting. As a result, such schemes may not provide a reliable indication of the accuracy of prediction models on unobserved data, thereby limiting their applications on predicting HPFRC properties. Moreover, existing data-driven ML models are often regarded as ‘black boxes’ with no explanation of their internal inference. In short, the application of conventional data-driven approaches to analyse HPFRC fracture behaviour may be ineffective due to the limited number of samples and the difficulty in conducting experiments. It is therefore essential to validate and comprehend the applicability of novel approaches, such as using interpretable modelling approaches for HPFRC.

In this context, sensitivity analysis has emerged as an advanced technique for gaining insights into the input-output relationships in prediction models. It measures the impact of input variables on the output by observing how the output changes with varying inputs. Two primary methods, known as local and global analysis, are employed for sensitivity analysis. The local approach is deemed suitable for linear models since it examines the effect of individual input variables by changing one variable at a time [27]. Local sensitivity analysis is preferred in various material studies due to its simplicity, minimal data requirements and low computation costs [26,[28], [29], [30]]. However, applying local sensitivity analysis to nonlinear models can sometimes lead to unreliable conclusions [27,31]. On the other hand, global approaches (such as the Sobol method) provide a more accurate and intricate analysis of nonlinear and non-additive models [32]. As such, global sensitivity analysis is deemed to be more suitable for evaluating ML-based prediction models incorporating human domain knowledge. Despite its advantages over local sensitivity analysis, global sensitivity analysis has not been effectively employed to interpret ML models using for characterising HPFRC.

This paper aims to address two major gaps in the analysis of HPFRC fracture behaviour at high strain rates: (a) the challenges associated with conducting experimental studies due to their expensive and complicated setups, and (b) the limitations in applying ML approaches to assess HPFRC fracture behaviour. A number of ML models (i.e., Random Forest, Extreme Gradient Boosting, Deep Neural Network, and Deep Residual Network) were implemented both with and without ensemble techniques. These models underwent training using small-scale datasets obtained from our prior experiments, as detailed in relevant experimental works [1,3,12,13,18]. Furthermore, a novel assessment method that combined data-based evaluation and human-involved evaluation was proposed in this study. Specifically, a hybrid procedure was developed in which the performance of derived prediction models was evaluated in two different ways (i) a conventional data-based approach (i.e., using error metrics such as coefficient of determination, root mean squared error and mean absolute error): under a K-fold cross-validation scheme; and (ii) a human-involved assessment approach with global sensitivity analysis. In the latter approach, domain experts evaluated the multivariate effects between input and output parameters in the derived prediction model. More importantly, the proposed interpretable modelling framework provides a powerful tool for proactive material failure analysis for HPFRC structures, offering numerous insights into nonlinear HPFRC fracture behaviour, particularly under high strain rates.

The remainder of this paper is organised as follows. Section 2 outlines the proposed methods for constructing HPFRC prediction models, both with and without ensemble techniques. This section also provides a detailed description of the hybrid validation approach using an interpretable machine learning approach. In Section 3, the findings obtained from applying these proposed methods to the analysis of HPFRC fracture behaviour is presented, together with a discussion on their applicability. Finally, Section 4 presents the key conclusions from this research, its limitations and discusses directions for future work.

## 2 Methodology

### 2.1 Data collection

**Fig. 1:**
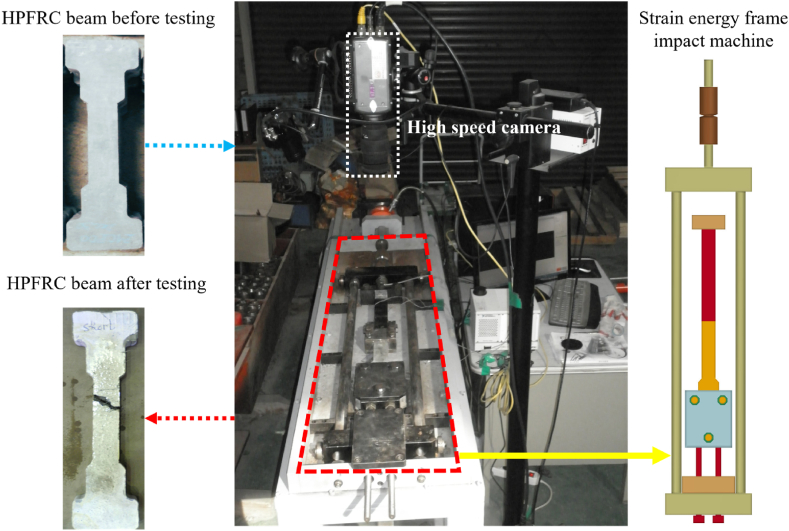
Test setup of HPFRC specimens with the Strain Energy Frame Impact Machine.

The HPFRC investigated in this study comprised high strength mortar matrix and various types of steel fibres. Specimen preparation and test setup are presented in Fig. 1. A strain energy frame impact machine (SEFIM) [18] was used to transfer tensile stress wave at high rate to the HPFRC beams by releasing strain energy in the frame. Tensile stresses were recorded by two strain gauges attached to the transmitter bar while displacements were obtained by a high-speed camera system. Additional information on the material components, mixture formulation, specimen preparation, and testing setup can be found in our previously published works [1,3,12,18].

ML-based modelling approaches were developed based on data obtained from laboratory experiments using SEFIM machine [1,3,12,18] to assess HPFRC fracture behaviour under high strain rates. A set of 147 datasets was collected from the aforementioned studies for training process of the proposed frameworks in Section 2. Each dataset includes eight independent variables representing physical and mechanical properties of fibre, mortar compressive strength, specimen's cross-sectional area and strain rate, and a dependent variable indicating fracture strength. Input variables include compressive strength of mortar matrix (M.Comp), specimen cross-sectional area (S.Cross), fibre diameter (F.Dia), fibre shape (F.Shape), fibre length (F.Length), fibre volume (F.Volume), fibre tensile strength (F.TStr) and strain rate (S.Rate). HPFRC beams were subjected to strain rates of up to 100 s^1^, with specimens having cross-sectional areas ranging ranging from 625 to 1250 mm^2^, composed of mortar with compressive strengths ranging from 56 to 180 MPa, and various types of fibre, including hooked, twisted, and short smooth shapes. Two input parameters, fibre density and modulus, are omitted from the proposed machine-learning-based model due to their constant setup in the experiments. Table 1 summarises the ranges of each variable included in this study.

**Table 1:** Description of input and output variables considered in this study.

Variable		Unit	Nomenclature	Investigated values
Input	Mortar compressive strength	MPa	M.Comp	[56−180]
	Specimen cross-sectional area	mm^2^	S.Cross	25 × 25, 25 × 50
	Fibre diameter	mm	F.Dia	[0.2–4.3]
	Fibre shape	–	F.Shape	Hooked, twisted, long smooth, short smooth
	Fibre length	mm	F.Length	[[13], [14], [15], [16], [17], [18], [21], [22], [23], [24], [25], [26], [27], [28], [29], [30], [31], [32]]
	Fibre volume	%	F.Volume	[1–1.5]
	Fibre tensile strength	MPa	F.TStr	[2311–2788]
	Strain rate	s^−1^	S.Rate	[0.000167–100]
Output	Fracture strength	MPa	–	[6.2–45]

### 2.2 Regression models for fracture strength of HPFRC at high strain rates

Multivariate regression models were used to assess HPFRC fracture behaviour subjected to high strain rates based on the datasets described in the preceding section. To model and analyse the relationships between the selected independent variables and the fracture strength, multiple machine learning techniques were employed as a class of regression analysis. The following subsections provide a brief mathematical background and description of the proposed approaches.

#### 2.2.1 Regression analysis

The modelling of HPFRC fracture strength was developed based on input variables including mortar strength, specimen cross-sectional area and fibre physical/mechanical properties along with strain rate. The problem of estimating fracture strength was formulated as a regression task, which involved learning an estimation function *f(x*_*1*_*,x*_*2*_*, …,x*_*n*_*)* mapping input variables *X={x*_*1*_*,x*_*2*_*, …,x*_*n*_*}* to the output *y*, i.e., fracture strength. Regression analysis infers the model of the independent input variables *X={x*_*1*_*,x*_*2*_*, …,x*_*n*_*}* and output dependent variable $\hat{y}$ (Eq. (1)), with the error term *e* representing the noise in observable data.

(1) $$\hat{y}=f\left(X\right)+e$$

The objective of regression analysis is to minimise the loss function $L\left(\hat{y}\approx f\left(X\right),y\right)$, where *y* represents the observed output. Commonly used loss functions for regression problems are the squared error and the absolute error functions (Eqs. (2), (3)) respectively).

(2) $$L\left(\hat{y},y\right)=\sum_{i}|y_{i}-f\left(X\right){|}^{2}$$

(3) $$L\left(\hat{y},y\right)=\sum_{i}|y_{i}-f\left(X\right)|$$

Regression analysis can provide forecasts and predictions based on recorded observations (*X*_*i*_*,y*_*i*_) by inferring the causal relationships between independent (input) variables and dependent (output) variables. Various methods, such as ML models, were investigated to optimise the representation for *f* by minimising the loss function L:

(4) $$f^{*}=\arg\min_{f}E_{\left(X,y\right)}L\left(y,f\left(X\right)\right)$$

where L is the loss function chosen based on the optimisation method, and *E*_(*X,y*)_ is the expectation over the entire set of independent variables *X* and dependent variable *y*.

#### 2.2.2 Machine learning-based prediction models

Conventional regression modelling of material properties has relied on ML techniques [22,23,28,[33], [34], [35], [36], [37], [38], [39]]. This strategy typically requires a substantial quantity of training and evaluation data. In this study, these approaches were extended by (i) employing a wide range of ML models to construct such data-driven ML models with limited data and (ii) proposing an advanced method for evaluating these models. A comprehensive evaluation of numerous ML models representing various ML method categories was conducted with (i) ensemble-based algorithms (Bootstrap Aggregating approach with Random Forests and Gradient Boosting approach with eXtreme Gradient Boosting) and (ii) feed-forward gradient-based neural networks (Deep Learning approach with Deep Neural Network and Deep Residual Neural Network).

**Random Forest:** Developed from Decision Tree [39,40], Random Forest (RF) [41] is a ML method using to build multiple independent trees to address the generalisation of bias problem. RF applies the Bootstrap Aggregating (Bagging) methods [42], involving the creation of multiple parallel independent trees and a discriminant function to combine the predictions of these trees while still maintaining their accuracy in making the prediction. The tree construction in the RF algorithm [43] is based on randomly selecting subspaces from the entire feature space. Each tree is built with the whole training data of corresponding selected features with the option with and without bootstrapping, i.e., drawing samples with and without replacement [43]. In this way, each tree follows its own optimisation paths with different sets of input features. The use of all these predictions from multiple trees, therefore, eliminates the bias of a single decision tree.

Utilising a discriminant function, the predictions generated by multiple decision trees are aggregated to yield a final outcome. Chou et al. [34], for instance, employed an averaging function to amalgamate the independent predictions from each individual tree within the "forest", in order to build a comprehensive model for high-performance concrete compressive strength:

(5) $$\hat{f}=\frac{1}{B}\sum_{b=1}^{B}f_{b}\left(X_{b}\right)$$

where *B* is the number of decision trees that the RF algorithm generates, *X*_*b*_ is the subset of input features used to build decision tree *b*, *f*_*b*_(*X*_*b*_) is the estimated output from decision tree *b* given input *X*_*b*_, and $\hat{f}$ is the bagging output for the RF algorithm.

**Extreme Gradient Boosting Algorithm:** In contrast to the Bagging approach [42], Friedman [44] proposed an alternative decision-tree solution in which multiple trees were sequentially constructed from the residuals of their predecessors. Chen and Guestrin [45] implemented eXtreme Gradient Boosting (XGBoost), which significantly enhanced system scalability and parallel out-of-core tree learning with regularisation. Typically, XGBoost uses an additive model with the addition of K weak prediction models *f*_*k*_(*X*_*i*_)|*k* = 1*..K* to accurately predict output *y* [44,45]:

(6) $${\hat{y}}_{i}=\varphi \left(X_{i}\right)=\sum_{k=1}^{K}f_{k}\left(X_{i}\right)$$

where (*X*_*i*_*,y*_*i*_) exemplifies the training data, and *f*_*k*_ represents the space of regression trees. Regularisation can also be incorporated into XGBoost objective function to prevent overfitting and reduce the model complexity [45]. The loss function is then adjusted accordingly to Eq. (7).

(7) $$L\left(\varphi \right)=\sum_{i}l\left({\hat{y}}_{i},y_{i}\right)+\sum_{k}\Omega \left(f_{k}\right)$$

where $\Omega \left(f\right)=\gamma T+\frac{1}{2}\lambda {\left\|\omega \right\|}^{2}$ with *T* representing the number of leaves in a tree *f*_*k*_, ω representing the weights associated with the *f*_*k*_ tree, and *l* representing the loss function of the difference between the target value *y*_*i*_ and its prediction ${\hat{y}}_{i}$. Using the assumptions that *t* is the iteration index and *f*_*t*_*(X*_*i*_*)* is the addition function at *t*-iteration ${\hat{y}}_{i}^{\left(t-1\right)}$*,* the loss function L at *t*-iteration is rewritten in Eq. (8) [45].

(8) $$L^{t}=\sum_{i}l\left(y_{i},{\hat{y}}_{i}^{\left(t-1\right)}+f_{t}\left(X_{i}\right)\right)+\Omega \left(f_{t}\right)$$

Chen and Guestrin [45] used a second-order approximation for *f*_*t*_*(.)* to ascertain the optimal weights for the tree:

(9) $$f_{t}\left(X_{i}\right)\approx g_{i}f_{t}\left(X_{i}\right)+\frac{1}{2}h_{i}f_{t}^{2}\left(X_{i}\right)$$

where $g_{i}=\partial_{{\hat{y}}^{\left(t-1\right)}}l\left(y_{i},{\hat{y}}^{\left(t-1\right)}\right)$ and $h_{i}=\partial_{{\hat{y}}^{\left(t-1\right)}}^{2}l\left(y_{i},{\hat{y}}^{\left(t-1\right)}\right)$.

The weights of the leaf *j*, *ω*_*j*_, is then optimised by considering the loss function $L^{t}\left(q\right)$:

(10) $$L^{t}\left(q\right)=-\frac{1}{2}\sum_{j=1}^{T}\frac{{\left(\sum_{i\in I_{j}}g_{i}\right)}^{2}}{\sum_{i\in I_{j}}h_{i}+\lambda }+\gamma T$$

where *q* represents the tree's structure, and *I*_*j*_ represents the instance set for the leaf *j*.

To improve the prediction accuracy, branches are added to the tree (by continuously split leaves) from candidate split points identified using the loss function in Eq. (11) [45]:

(11) $$L_{split}=-\frac{1}{2}\left[\frac{{\left(\sum_{i\in I_{L}}g_{i}\right)}^{2}}{\sum_{i\in I_{L}}h_{i}+\lambda }+\frac{{\left(\sum_{i\in I_{R}}g_{i}\right)}^{2}}{\sum_{i\in I_{R}}h_{i}+\lambda }-\frac{{\left(\sum_{i\in I}g_{i}\right)}^{2}}{\sum_{i\in I}h_{i}+\lambda }\right]+\gamma$$

where *I* is the instance set of all nodes, while *I*_*L*_ and *I*_*R*_ are the instance sets of left and right nodes respectively.

**Deep Neural Network.** Deep Neural Network (DNN), also known as Deep Feed Forward Network, constitutes a deep learning model characterised by the presence of numerous layers of perceptron. DNN models the mapping *y* = *f*(*x*;*θ*), where *x* is the input features, *y* is the output, and *θ* represents the model's parameters. In DNN models, information flows in a forward direction, from the first layer to the final layer without any feedback links [46].

**Fig. 2:**
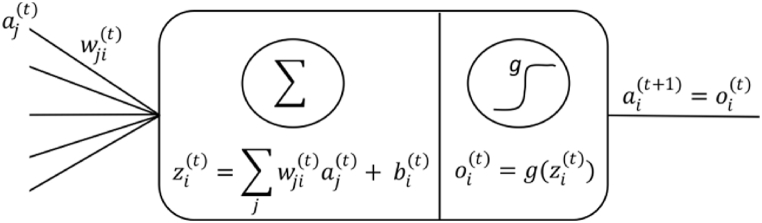
Anatomy of the i-th processing unit in layer t, depicting inputs from units in the previous layer (t-1) and output to the next layer (t+1).

DNN comprises multiple processing units, or single perceptron, characterised by three essential components: weights (w), bias (b), and activation function (*g(.)*). Fig. 2 depicts an example of a conventional processing unit in a DNN model. Denoting that $a_{j}^{\left(t\right)}$ is an output value of the *j*-th unit in the previous layer (*t*-1), $w_{ji}^{\left(t\right)}$ is the weight coefficient associated with the *j*-th input and the *i*-th unit in the layer *t*, and *g(.)* is the activation function of the unit, the output of the described processing unit is calculated following Eq. (12):

(12) $$a_{i}^{\left(t+1\right)}=g\left(\sum_{j}w_{ji}^{\left(t\right)}a_{j}^{\left(t\right)}+b_{i}^{\left(t\right)}\right)$$

**Fig. 3:**
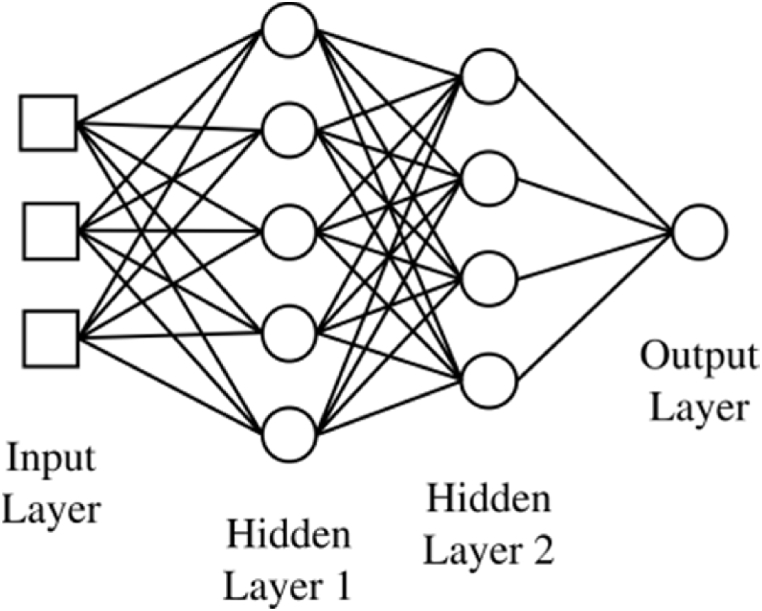
A 3-layer network architecture depicting each processing node described in Fig. 2. The leftmost layer, comprising input features, is referred to as the input layer. The two intermediate layers, positioned in the middle and containing network values, are denoted as hidden layers. The rightmost layer is designated as the output layer.

The network structure of DNN is constructed by interconnecting multiple layers, where each layer consists of multiple processing units. In the specific case of a 3-layer network structure depicted in Fig. 3, the output is computed in matrix form using Eq. (13).

(13) $$\hat{Y}=g\left(W^{\left(2\right)T}A^{\left(2\right)}+b^{\left(2\right)}\right)=g\left(W^{\left(2\right)T}g\left(W^{\left(1\right)T}A^{\left(1\right)}+b^{\left(1\right)}\right)+b^{\left(2\right)}\right)=g\left(W^{\left(2\right)T}g\left(W^{\left(1\right)T}X+b^{\left(1\right)}\right)+b^{\left(2\right)}\right)$$

where *g(.)* is an element-wise activation function, *W*^(*t*)*T*^ is the transposed weight matrix for layer *t*, *b*^(*t*)^ is the bias terms associated with layer *t*, *X* is the input vector, and $\hat{Y}$ is the prediction output of DNN.

To optimise the performance of DNN, the backpropagation algorithm and chain rule [47] were used to compute the gradient of coefficients (weights and biases) for each processing unit. These coefficients were then updated using the gradient descent method [48,49]. For regression problems, the backpropagation loss function is typically the squared error, as defined by Eq. (14).

(14) $$J\left(\theta \right)=\frac{1}{2}{\left\|Y-\hat{Y}\right\|}^{2}$$

Multiple methods were proposed to improve the performance of DNN, such as increasing the network's complexity [46,50], applying regularisation methods [[51], [52], [53]], employing normalisation techniques [54], or substituting canonical gradient descent with other optimisation methods [[55], [56], [57]].

Despite DNN being widely applied to address numerous regression and classification problems, it is susceptible to the issue of gradient vanishing, a challenge shared by other network structures trained with the backpropagation algorithm [58,59]. Several proposed solutions to this problem include unsupervised pre-training [60], using dropout [51], utilising sigmoid-alternative activation functions such as a rectified linear function [61], or employing deep residual networks [36,62].

**Fig. 4:**
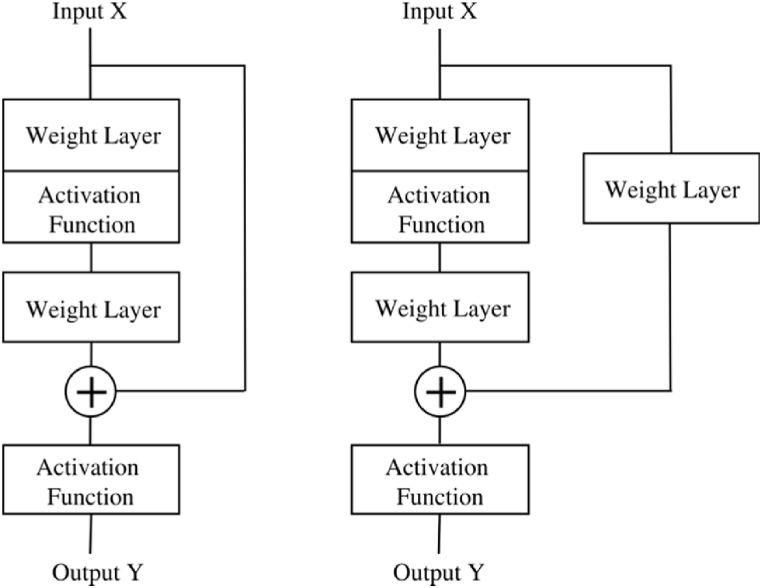
(Left) A simple ResNet block structure comprising 2 wt layers and an identity shortcut connection from input to output. (Right) A generalised ResNet block structure featuring an additional weight layer to ensure matching of input and output dimensions.

Deep Residual Network. Deep residual networks (ResNet) [62] have demonstrated their effectiveness in addressing the persistent challenge of gradient vanishing in DNN, particularly when dealing with increased network complexity [59]. The key to this improvement lies in the incorporation of shortcut links between layers within the network architecture [62], enabling the neural network to proficiently learn novel mapping functions. Specifically, Fig. 4 (left) presents a simplified ResNet configuration featuring an identity shortcut originating from the input. Without the skip connection, Fig. 4 closely resembles a two-hidden-layer DNN aiming to learn the mapping function *H(X)*. By employing the skip connection in ResNet, *H(X)* can be partitioned into two distinct components: *X* (from the skip connection) and *F(X)* (a mapping function formed by the two weight layers), as depicted in Eq. (15), assuming that the input and output dimensions are identical.

(15) H(X)=F(X)+X

He et al. [62] proposed a generalised formulation (derived from Eq. (15)) for the ResNet structure, which accounts for discrepancies in input and output dimensions (see Eq. (16)). In Fig. 4 (right), an additional layer is incorporated to ensure alignment between the dimensions of the skip connection and the output of *F(X)* before their summation [36].

(16) $$H\left(X\right)=F\left(X\right)+W_{s}X$$

Nguyen et al. [36] showcased the enhanced performance of ResNet in systems featuring network structures comparable to DNN. In addition, some variants of ResNet architecture for regression problems were also presented, incorporating standard implementations of dropout and regularisation techniques into the ResNet framework.

#### 2.2.3 Hybrid evaluation method for fracture strength prediction models

This section introduces a hybrid approach to evaluate prediction models for HPFRC fracture strength under high strain rates. The approach encompassed two evaluation methods: a data-based method and a sensitivity-analysis-based method. The data-based method employed a validation set of data to quantify the error between the predicted and target values. Sensitivity-analysis-based method utilised global sensitivity analysis to explore the relationship between independent input and dependent output features. This method allowed interpretation of the models and their validation using empirical knowledge obtained from experiments or domain experts. The combination of both methods provides a more comprehensive evaluation of the model's accuracy and reliability, especially for models derived from limited experimental data.

##### 2.2.3.1 Data-based evaluation method

In order to evaluate the accuracy of the proposed models using data-based evaluation method, multiple error measures were employed to compare the predicted output values generated by the models with the target values obtained from physical experiments in the prepared datasets. Statistical error metrics, including the coefficient of determination (R^2^), root mean square error (RMSE), and mean absolute error (MAE), were utilised to assess the performance of the prediction models.

To ensure proper evaluation and avoid overfitting models, data-based assessment of regression models requires random division of the original dataset into training and evaluation datasets. It is important to note that for small datasets, the arrangement of data samples into training and testing sets has a significant impact on the statistical error metrics. In such cases, the utilisation of the K-fold cross-validation method may be necessary to account for the randomness in testing model performance, considering the limitations posed by the size of the data.

**Error metrics:** Three commonly used error metrics, namely R^2^, RMSE, and MAE, were utilised to assess the accuracy of regression models in predicting fracture strength (Eq. (17), (18), (19))). These metrics have a well-established presence in the literature for evaluating the performance of models in predicting concrete's properties.

(17) $$R^{2}=1-\frac{\sum_{i}{\left(y_{i}-\hat{y_{i}}\right)}^{2}}{\sum_{i}{\left(y_{i}-\bar{y}\right)}^{2}}$$

(18) $$RMSE=\sqrt{\frac{1}{n}\sum_{i=1}^{n}{\left(y_{i}-{\hat{y}}_{i}\right)}^{2}}$$

(19) $$MAE=\frac{1}{n}\sum_{i=1}^{n}|y_{i}-{\hat{y}}_{i}|$$

where *n* is the sample size, *y*_*i*_ is the observed output value of the *i*-th sample, ${\hat{y}}_{i}$ is the predicted output value of the *i*-th sample, and $\bar{y}=\frac{1}{n}\sum_{j=1}^{n}y_{j}$ is the mean of observed output values.

**Fig. 5:**
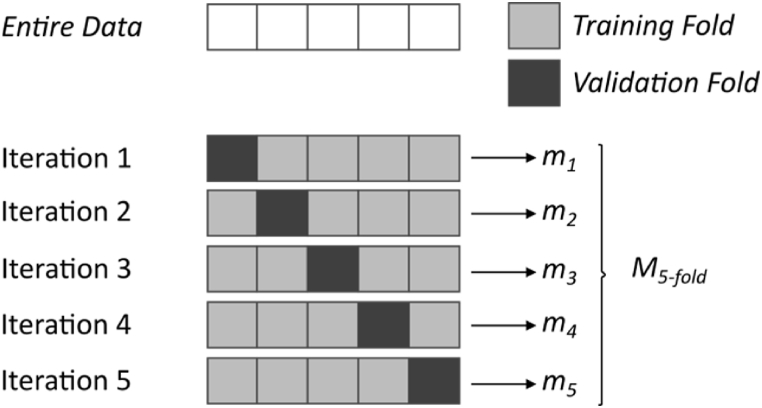
An example of *K-fold* cross-validation scheme with *K = 5*.

**K-fold cross-validation scheme:** The K-fold cross-validation method involves dividing the original dataset into *K* folds and performing *K* independent training iterations with *(K-1)* folds used for training various prediction models, and the last fold reserved for validation. Fig. 5 illustrates a fundamental example of the K-fold cross-validation scheme. In this study, *K=10* was chosen as the number of folds. Average values of R^2^, RMSE, and MAE obtained from K-training and evaluation iterations were used to assess the performance of prediction models. The evaluation of the prediction models' performance was carried out using the following equation:

(20) $$M_{K-fold}=\frac{1}{K}\sum_{k=1}^{K}m_{k}$$

where *M*_*K−fold*_ denotes a general metric measurement when K-fold cross validation scheme is applied, and *m*_*k*_ is the metric measurement in the *k*-th iteration of the procedure.

##### 2.2.3.2 Global sensitivity analysis-based evaluation method

In addition to data-based evaluation method, a global sensitivity analysis approach was also developed to further evaluate the performance of HPFRC fracture strength. This method allows for a more comprehensive investigation of the effects of individual independent variables, as well as their interactions, on the dependent variable compared to local analyses. The Sobol sensitivity analysis [32] was chosen as a typical global sensitivity analysis as it offers a model-agnostic strategy treating prediction models as black boxes with a general mapping function of *Y = f(X),* where *X = {x*_*1*_*,x*_*2*_*, …,x*_*d*_*}.* In this approach, the variance of *f(.)* is decomposed as shown in Eq. (21) [63]:

(21) $$Var\left(Y\right)=\sum_{i=1}^{d}D_{i}\left(Y\right)+\sum_{i<j}^{d}D_{ij}\left(Y\right)+...+D_{123..d}\left(Y\right)$$

where Var(Y) is the variance of Y=f(X), $D_{i}\left(Y\right)=Var\left(E\left[Y|x_{i}\right]\right)$, $D_{ij}\left(Y\right)=Var\left(E\left[Y|X_{i},X_{j}\right]\right)-D_{i}\left(Y\right)-D_{j}\left(Y\right)$ and similarly for other higher-order components of Var*(Y)*. Sobol variance-based indices are measured based on Eq. (22) [63]:

(22) $$S_{i}=\frac{D_{i}\left(Y\right)}{Var\left(Y\right)},S_{ij}=\frac{D_{ij}\left(Y\right)}{Var\left(Y\right)},...$$

The total effects of an input feature *i* to the variance of the output is then calculated in Eq. (23):

(23) $$S_{T_{i}}=\sum_{l\subset \#i}S_{l}$$

where *#i* denotes all subsets of *{1,2,3,..,d}* containing *i*. Sobol method [32] also includes a Monte-Carlo approximation approach for calculating Sobol indices, which estimates Sobol indices based on the model's response to given inputs according to a predetermined scheme.

While local sensitivity analysis has been commonly used in developing prediction models for concrete materials [26,[28], [29], [30]], its limitations in assessing nonlinear models have been reported in several studies [27,31]. In contrast, global sensitivity analysis, such as the Sobol method [32], offers a more comprehensive and accurate analysis of nonlinear models. This approach proves particularly valuable in validating machine learning-based prediction models by incorporating human domain knowledge. However, despite the potential advantages of global sensitivity analysis in the application of machine learning models to concrete, its full potential remains underutilised, necessitating further investigation to fully harness its capabilities.

#### 2.2.4 Learning framework for fracture strength assessment

The proposed framework for constructing and validating regression models to assess HPFRC fracture strength comprises four primary steps: (i) collecting and pre-processing data from experiments; (ii) selecting the most effective learning technique using the K-fold cross-validation scheme and data-based evaluation methods; (iii) interpreting the models from step (ii) using sensitivity analysis and expert validation; and (iv) exploring the possibility of integrating an ensemble method with K model instances from step (ii). Step (iii) can also be used to identify interesting input-output relationships for further experimental investigation.

##### 2.2.4.1 Framework for machine learning model selection

The application of ML models on predicting material mechanical properties has been explored by previous studies in the current literature. Yeh [33] used artificial neural network (ANN) and 4-fold cross-validation method to model compressive strength of high-performance concrete (HPC). Chou et al. [34,35,64] investigated different data-mining techniques to improve the accuracy of HPC compressive strength models, using a 10-fold cross-validation strategy to evaluate the regression models. Other studies [23,28,37,65] trained various ML models using a simple training/testing data split or a K-fold cross-validation scheme to minimise the loss function between predicted and target values of the dependent variable. Although these models were evaluated with different error metrics, only a few models underwent sensitivity analysis after data-based evaluation phase [28].

**Fig. 6:**
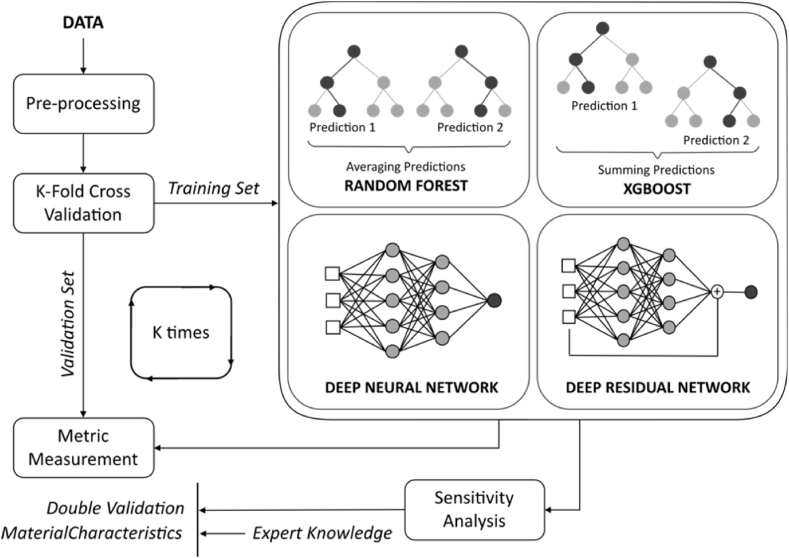
Framework for machine learning model selection in predicting high strain rate behaviour of HPFRC.

To mitigate the risk of inferring spurious correlations between independent and dependent variables in datasets with limited sample sizes, a hybrid evaluation method was employed to assess the performance of learned prediction models. Fig. 6 depicts the proposed framework for comparing and selecting the optimal prediction model using diverse ML techniques. Notably, an additional phase encompassing global sensitivity analysis and validation based on empirical knowledge extracted from experiments was incorporated into the K-fold cross-validation scheme to evaluate prediction models alongside error metric measurements.

The proposed framework facilitated the comparison and selection process of prediction models using various ML techniques, augmented by domain-specific expertise and global sensitivity analysis. While ML techniques excel at capturing complex and non-linear relationships between inputs and outputs, a notable drawback is the lack of interpretability in the resulting models. In addition, the scarcity of validation data in laboratory-based studies, particularly for HPFRC under high strain rates, due to costly and lengthy preparation of specimens has raised concerns about the reliability and applicability of ML models. To address these concerns, the suggested framework incorporated a hybrid validation procedure including global sensitivity analysis (i.e. Sobol sensitivity analysis) to comprehensively investigate the impact of inputs and their interactions on the output.

One objective of the proposed framework is to address the prevalent data limitations in experimental domains and establish interpretable relationships between inputs and outputs, thereby enhancing confidence in the utilisation of ML-based "black-box" prediction models. In situations where peculiar interactions among input variables are detected, targeted and efficient supplementary experiments may be suggested to elucidate these relationships and ensure the validity of the models.

##### 2.2.4.2 An ensemble approach to improve machine learning-based models

**Fig. 7:**
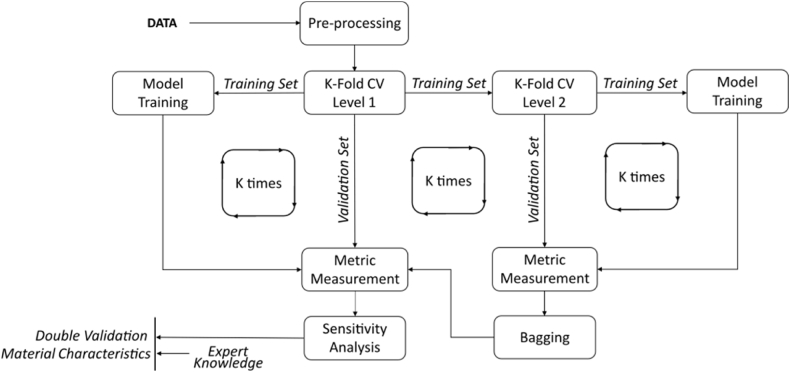
Framework to compare 2 directions: (i) optimising the model using the entire dataset, and (ii) bootstrapping K model instances from a K-fold cross-validation scheme.

In the final step, upon selecting the ML algorithms for modelling fracture behaviour, the possibility of further improving the performance of the selected model was considered by examining two approaches: (i) optimising the model using the entire dataset (referred to as "Train-All"), and (ii) constructing a K-fold ensemble model by bootstrapping K model instances from a K-fold cross-validation scheme (referred to as "K-fold Ensemble"). Fig. 7 presents a framework with two hierarchical levels of K-fold cross-validation for generating and comparing these models. Specifically, the first K-fold scheme (Level 1) compares the performance of the model trained with all available data to the ensemble model created by bootstrapping all model instances from the second K-fold scheme. The model instances from the second K-fold scheme (Level 2), obtained with an early-stopping feature based on RMSE measurement, are combined using bagging technique [42,44] to generate the K-fold ensemble model.

## 3 Results and discussion

This section provides an analysis and interpretation of various ML models applied for predicting HPFRC fracture strength using four ML approaches, including ensemble-based methods represented by RF and XGBoost, and feed-forward gradient-based neural network solutions represented by DNN and ResNet. These ML models were configured using diverse settings, and the ensemble technique was used to improve their prediction performance. Several additional trials with extended results are presented in Appendix A. To assess the trials, a hybrid validation process that combined data-based evaluation with global sensitivity analysis-based evaluation was utilised. The data-based evaluation allowed for a quantitative assessment of the prediction performance of the derived ‘black-box’ models from ML modelling approaches. Due to the limited sample size in experimental fields, global sensitivity analysis, particularly Sobol scoring, was utilised in this study to further qualitatively assess and analyse prediction models. It is important to note that this evaluation method focused on assessing the model itself, rather than examining the relationships between the inputs and the output from collected datasets.

### 3.1 Data-based validation for fracture strength prediction models

**Fig. 8:**
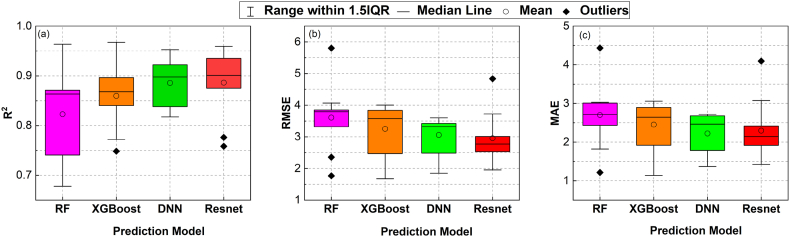
Boxplots for data-based evaluation results for the four selected models: RF, XGBoost, DNN and ResNet under different error measures: (a) R^2^, (b) RMSE and (c) MAE. The Interquartile Range (IQR) in the boxplots represents the middle 50 % of the accuracy measures of the models, spanning from the 25th to the 75th percentiles.

To assess the performance of ML approaches in modelling HPFRC fracture strength subjected to high strain rates, a 10-fold cross-validation scheme was utilised to eliminate biases produced by splitting the training and evaluation sets. The error metrics, including R^2^, RMSE, and MAE, were calculated on different evaluation sets under the K-fold cross-validation scheme, and the results are displayed in the boxplots of Fig. 8. The length of each box in Fig. 8 represents the interquartile interval, illustrating the range of measurement results obtained from a K-fold cross-validation scheme, spanning from the 25 % quartile to the 75 % quartile. The feed-forward gradient-based neural network group, represented by DNN and ResNet, demonstrated superior prediction ability on HPFRC fracture strength compared to ensemble-based techniques such as RF and XGBoost. However, the obtained error metrics were found to vary among folds in all models, indicating significant impact of limited datasets and biases in selecting data for training and evaluation sets on the derivation of prediction models. These results verify the necessity of using a K-fold cross-validation scheme when testing models with insufficient datasets. In other words, simple random splitting for training and validation sets is not an appropriate method for evaluating models with limited datasets.

As shown, the ResNet model (referred to as configuration 6 in Table A1 in appendix A) had the lowest average RMSE/MAE (RMSE = 2.956; MAE = 2.297) and the highest average R^2^ = 0.886, making it the most accurate model for predicting fracture strength. This model also demonstrated a more stable and consistent performance in all K-fold iterations compared to the others, as evidenced by its smaller interquartile ranges for all metric measures (Fig. 8).

**Fig. 9:**
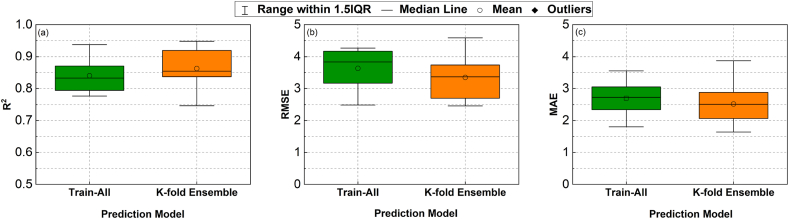
Data-based evaluation results of the two implementation approaches for ResNet models: (i) Using all the data to train the neural network ("Train-All") and (ii) Bootstrapping by combining K model instances from a K-fold cross-validation scheme ("K-fold Ensemble").

The results shown in Fig. 8 demonstrate the effectiveness of the ResNet model in learning the mapping between input features and HPFRC fracture strength. This modelling approach was selected to be considered in constructing the final models, referred to as "Train-All" and "K-fold Ensemble." A boxplot comparison of these implementation options is shown in Fig. 9. In training and testing these models, "K-fold Ensemble" outperformed "Train-All" with the exact data folds. Mean values of error metrics with the "K-fold Ensemble" model were R^2^ = 0.863, RMSE = 3.350, and MAE = 2.519, which were higher compared to those with "Train-All" model (R^2^ = 0.840, RMSE = 3.631, and MAE = 2.699). This result highlights the effectiveness of using a combined model constructed with distinct subsets of data, similar to the method employed by RF and XGBoost.

The sample size for each fold was smaller than those split in Fig. 9 because these comparisons relied on the testing method with two K-fold cross-validation schemes, as shown in Fig. 7. Therefore, the error metrics between Fig. 8, Fig. 9 cannot be compared. Instead, both schemes can be considered two crucial steps to the proposed strategy.

The accuracy of the proposed model, “K-Fold Ensemble” with the better performance compared to “Train-All”, was compared to other ML models in previous studies as shown in Table 2. Since the application of ML models in the literature of UHPFRC fracture behaviour under high strain rate is scarce, there is insufficient numbers of articles with similar topics. Therefore, ML models used to predict different types of mechanical properties, including tensile and flexural strength, reported in previous studies [[66], [67], [68], [69], [70], [71]] were considered for comparison purposes. As shown, the proposed model “K-Fold Ensemble” presented better performance than other ML models for predicting UHPFRC tensile strength including ANN and CBR with small-sized samples of up to 157 dataset Ramezansefat [67], with gain in percentage of up to 425.7 %. Regarding the studies with more datasets [[68], [69], [70], [71]], “K-Fold Ensemble” model showed moderate positive outcome with higher R^2^ compared to ANN, SVM and GPR models (a gain of 4.3 %–15 %), and lower accuracy (up to 9.9 %) than other ML models including XGBoost, SVR and GB. This observation can be explained by the fact that these studies [[66], [67], [68], [69], [70], [71]] only assessed tensile and flexural behaviours of various fibre-reinforced concrete types under normal loading condition with greater data availability. Characterising fracture behaviour of brittle materials such as HPFRC at high strain rate is deemed to be more challenging due to the complexity of testing setup to satisfy stress equilibrium requirements [72], leading to a scarcity of data needed for ML model development. Thus, the prediction accuracy of the proposed ML model is deemed to be acceptable for estimating HPFRC fracture strength at high strain rate.

**Table 2:** Performance comparisons with previous studies [[66], [67], [68], [69], [70], [71]].

References	Prediction models	Material properties	Material type	Numbers of datasets	R^2^	Gain in (%)
Khosravani et al. [66]	CBR	Tensile strength	UHPFRC	55	0.234 − 0.702	22.9 − 268.7
Ramezansefat et al. [67]	ANN	Tensile strength	Fibre reinforced concrete	157	0.006 − 0.758	13.8 − 425.7
Khokhar et al. [68]	ANN, SVM, CART, XGBoost, GPR	Tensile strength	Fibre reinforced concrete	438	0.740 − 0.950	−9.2 − 16.6
Guo et al. [69]	ANN, SVR, CART, XGBoost	Tensile strength	UHPFRC	387	0.827 − 0.957	−9.9 − 4.3
Qian et al. [70]	SVM, MLP, GB	Flexural strength	UHPFRC	317	0.710 − 0.910	−5.2 − 21.5
Kulasooriya et al. [71]	DT, GB, LGB	Flexural strength	Basalt fibre reinforced concrete	–	0.803 − 0.893	−2.2 − 7.4

Note: ANN = Artificial Neural Networks; SVM = Support Vector Machine; CART = Classification And Regression Tree; GPR = Gaussian process of regression; MLP = Multi-Layer Perceptron; GB = Gradient Boosting; DT = Decision Tree; LGB = Light Gradient Boosting; SVR = Support Vector Regression; CBR = Case-based Reasoning.

### 3.2 Global sensitivity analysis-based evaluation of fracture strength prediction models

**Fig. 10:**
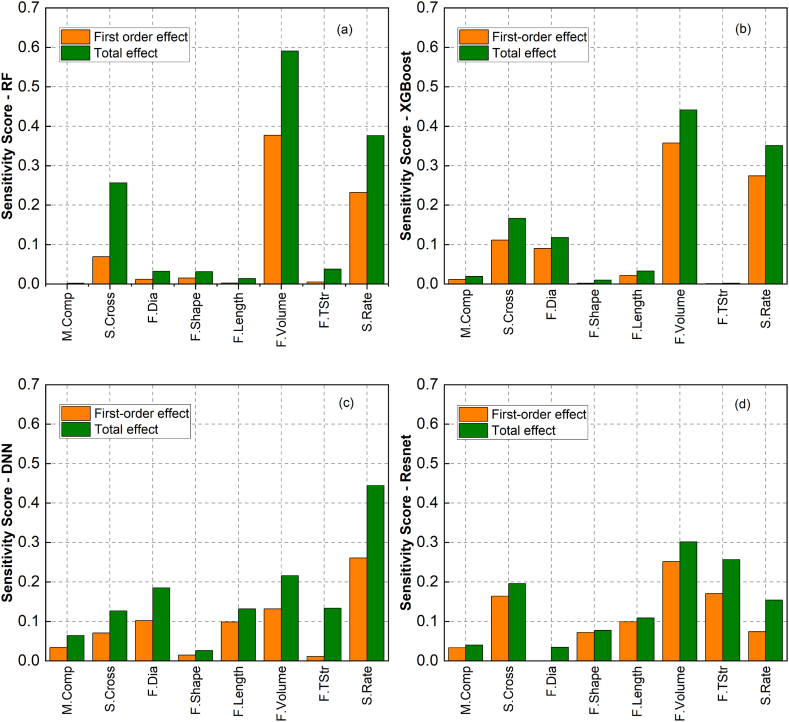
First-order and total effect sensitivity scores of models trained using various data-driven methods: (a) RF, (b) XGBoost, (c) DNN and (d) ResNet.

To increase the validity of the derived ML prediction models, global sensitivity analysis and human domain expertise were employed to further validate these models. Fig. 10 illustrates the contribution of each input variable to two scopes - first-order effect and total effect - for the four prediction models. The first-order effect indicates the effect of each input on output without considering the interaction between inputs, while the total effect identifies the impact of each input on output, considering the interaction between inputs. This study considered eight selected input variables, including concrete mortar compressive strength, specimen cross-sectional area, fibre diameter, fibre shape, fibre length, fibre volume, fibre tensile strength and strain rate.

In Fig. 10 (a), the RF model indicates that the compressive strength of cementitious mortar and fibre characteristics had minor roles in explaining the output variation, while the most influential factors on HPFRC fracture strength were fibre volume, strain rate, and specimen cross-sectional area. The sensitivity score of their first-order effects indicated that roughly 67.9 % of the output variation can be explained by first-order input variation, with fibre volume having the highest score at 37.7 %. Considering interactions between inputs, the total-effect score for fibre volume was approximately 59.1 %, followed by strain rate (37.7 %) and specimen cross-sectional area (25.5 %). Similarly, for the XGBoost model, Fig. 10 (b) shows high sensitivity scores ranging from 11.1 % to 35.7 % for the first-order effect and from 16.7 % to 44.2 % for the total effect, indicating the significance of the same set of input variables (i.e., fibre volume, strain rate, and specimen cross-sectional area) in explaining output variation. The remainder of the inputs in XGBoost model had negligible effects on predicting HPFRC fracture strength, similar to RF model.

In contrast to RF and XGBoost models, feed-forward gradient-based models involved more inputs in configuring the model, resulting in a more uniformed distribution of input's impact on the output. In DNN model, except for strain rate, which has the highest sensitivity score (26.1 % for the first-order effect and 44.4 % for the total effect), the remaining inputs, including fibre shape and mortar compressive strength were the least influential parameters with the lowest first-order and total effect scores ranging from 1.5 % to 3.4 % and 2.6 %–6.4 %, respectively. ResNet model identified the contribution from most inputs including fibre volume and properties, specimen cross-sectional area and strain rate with sensitivity scores ranging from 7.3 % to 26.1 % and 7.9 %–32.2 % for the first-order and total effects, respectively. Fibre volume was the most critical variable in explaining output variation with the highest sensitivity scores. Unlike other models, the effect of fibre shape on HPFRC fracture strength obtained in ResNet model was not generally negligible with total sensitivity score of 7.9 %, thus varying fibre geometry in the specimens might cause variations in their fracture behaviour. This observation is in agreement with other experimental studies [[73], [74], [75]].

**Fig. 11:**
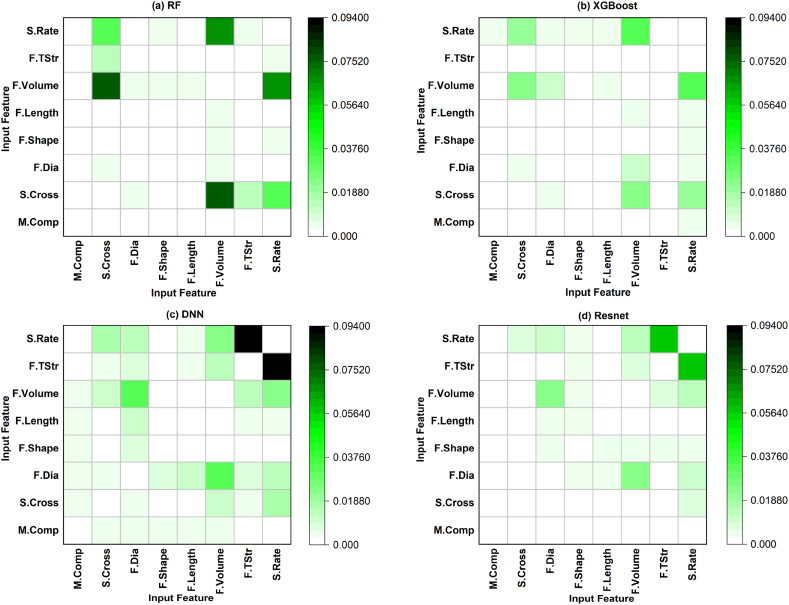
Second-order sensitivity scores obtained from Sobol sensitivity analysis for: (a) RF, (b) XGBoost, (c) DNN and (d) ResNet models.

The second-order sensitivity analysis results for all machine learning models are presented in Fig. 11 as a heat map, which illustrates the interaction between pairs of eight input parameters. They highlighted the impact of one factor on another for the investigated inputs investigated. For both RF and XGBoost models, the input pairs (F.Volume, S.Cross) and (F.Volume, S.Rate) played the dominant role among all the pairs, respectively, as shown in Fig. 11(a and b). In contrast, the pair (S.Rate, F.TStr) was the most influential pair for both DNN and ResNet models, with a second-order score of up to 0.094 (or 9.4 % in output variation), demonstrating that their interaction was important for controlling the variability of fracture strength. The consistency of DNN and ResNet was then highlighted in comparison to RF and XGBoost models in fracture strength prediction. The pronounced effect of high strain rate on HPFRC fracture behaviour was also observed, in line with expectations, as both concrete and steel are known to be strain-rate sensitive materials [76]. Unlike limited scope in previous studies [1,3,6,73], the obtained results also revealed the interaction between various input factors such as strain rate and fibre tensile strength. The input pair (S.Rate, F.TStr) with a second-order score of 0.057 (or 5.7 % in output variation) was important in controlling the variability of fracture strength. Additionally, the mutual effect between the pair (F.Volume, F.Dia) in DNN and ResNet models, with a sensitivity score of up to 0.032 (or 3.2 % in output variation), was also detected significant for HFPRC fracture behaviour.

Sensitivity scores from total effect measurements indicated that strain rate, fibre volume, and specimen cross-sectional area were the most influential inputs for modelling HPFRC fracture strength in the four selected models. This result was consistent with previous experimental studies [12,[77], [78], [79]] employing SIFIM to investigate HPFRC fracture behaviour subjected to high strain rates. These studies discovered that a higher fracture strength associated with a higher strain rate due to rate-sensitive interfacial bond strength between mortar matrix and fibre, as well as the physical and mechanical properties of fibres (i.e., shape, length and tensile strength). The alignment of the findings obtained from this study with those studies supports the proposed approach of integrating ML and global sensitivity analysis for small-scale datasets.

**Fig. 12:**
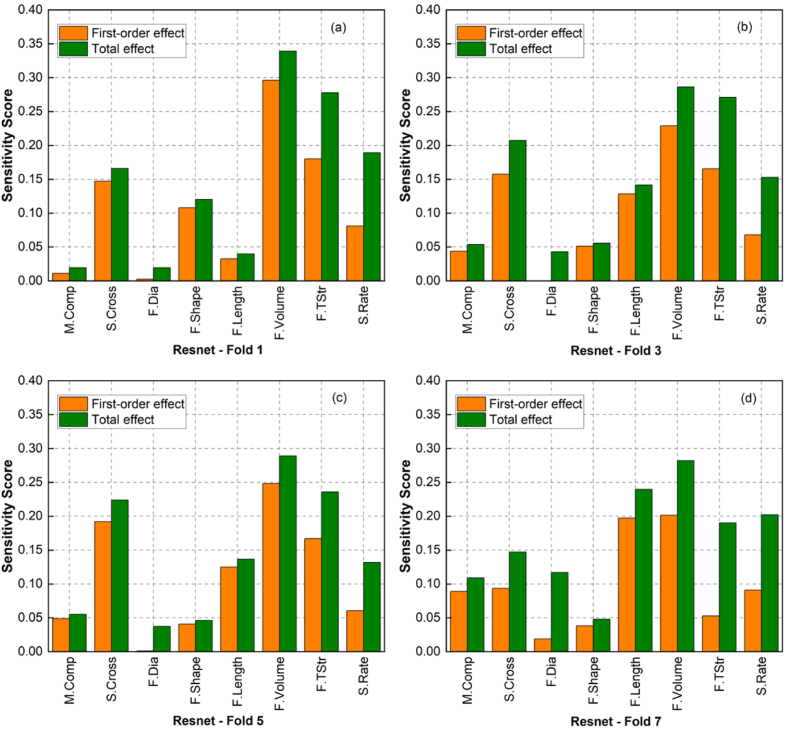
First-order-effect and total-effect sensitivity scores for Resnet models trained under K-fold cross-validation scheme: (a) Fold 1, (b) Fold 3, (c) Fold 5 and (d) Fold 7.

**Fig. 13:**
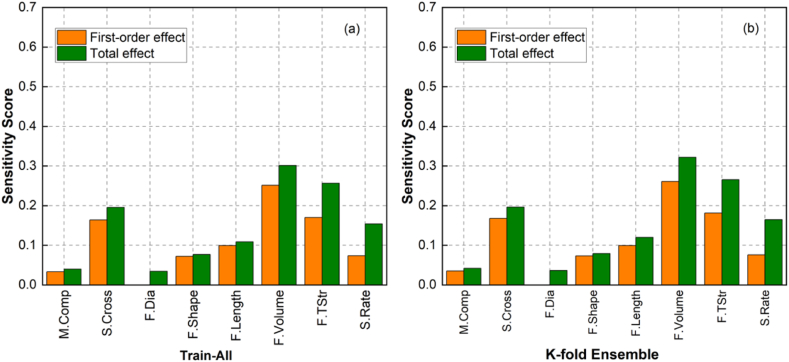
First-order and total effect sensitivity scores for Resnet models: (a) Train-All and (b) K-fold Ensemble.

From the validation process using global sensitivity analysis, the ML modelling approach leveraging ResNet architecture was considered the most effective method for modelling HPFRC fracture behaviour due to its high reliability in terms of statistical error metrics (Fig. 8) and its interpretation of input factors (from sensitivity analysis results in Fig. 10). Validation results for the ResNet model, demonstrating the best error metrics on the validation sets using the K-fold cross-validation scheme, are presented in Fig. 12. In addition, sensitivity scores for the first-order and total effects of ResNet models trained in the first, third, fifth, and seventh K-fold iterations were included in this figure. The sensitivity scores for the "Train-All" and "K-fold Ensemble" models are shown in Fig. 13, following the process outlined in Fig. 7. The alignment of the results across multiple models and validation methods strengthened the reliability and robustness of this ML modelling approach.

As shown, fibre volume, tensile strength, strain rate, and specimen cross-sectional area remained the most critical input features. The sensitivity score distribution of the "K-fold Ensemble" model presented in Fig. 13 (a) closely resembled that of "Train-All" model in Fig. 13 (b), derived from the training procedure with all available data. Both of these models demonstrated that all input features significantly contribute to output variation, i.e., fracture strength. In contrast to ResNet models presented in Fig. 12, "Train-All" and "K-fold Ensemble" models consistently presented the influence of fibre physical properties (i.e., shape, length, and diameter) on fracture strength. This result is consistent with other experimental studies [12,[77], [78], [79]], in which HPFRC fracture strength was highly sensitive to these variables. The fact that the sensitivity analysis of the proposed ML models matches those of expert-designed experiments consolidated the confidence in employing the ensemble models derived from the method illustrated in Fig. 7.

## 4 Conclusion

This study proposed a novel framework for modelling HPFRC fracture behaviour subjected to high strain rates ranging from 0.000167 to 100 s^−1^ by utilising interpretable machine learning modelling approaches. The following highlights a summary of the findings:

• Among the four approaches, ResNet model demonstrated the highest performance with the lowest average RMSE/MAE and the highest average R^2^ (R^2^ = 0.886, RMSE = 2.956 and MAE = 2.297). Therefore, this model was selected to construct the final models, "Train-All" and "K-fold Ensemble”, in which the later outperformed the former with R^2^ = 0.863, RMSE = 3.350, and MAE = 2.519.
• Deviations in data-based evaluation results for derived machine learning models were observed across different K-fold iterations, which highlighted the challenges associated with training and evaluating modes with limited experimental data. The proposed framework, which incorporates data-driven approaches and Sobol global sensitivity analysis, provided insights into the construction and validation of prediction models in such situations. In addition, the hybrid evaluation procedure and data-driven modelling approaches can be adapted to similar tasks associated with small dataset collections.
• HPFRC fracture strength were significantly influenced by various input factors, including fibre volume and mechanical/physical properties, strain rate, specimen cross-sectional area, and their interactions with other inputs.
• HPFRC specimens were sensitive to various input pairs including strain rate – fibre tensile strength and fibre volume – fibre diameter. To prevent fracture failure, the inclusion of high tensile strength fibres correspondingly to strain rate or adjusting fibre dosage and its physical properties in HPFRC is recommended.

The proposed framework is deemed to serve as a valuable starting point for improving both accuracy and reliability of ML models performance in predicting HPFRC mechanical properties, especially when confronted with limited datasets from laboratory-based experiments. The integration of human expert feedback proves crucial, underscoring its significance relative to dataset size. The necessity of validating the model through both a data-based approach (utilising error metrics) and a global sensitivity analysis-based approach (including validation from domain experts) was emphasised through the obtained findings. The final model, built based on the Resnet architecture and K-fold Ensemble approach, was shown to meet both these validation criteria.

## 5 Limitation and future research

The proposed interpretable framework demonstrates the capability to model mechanical characterisation of HPFRC under high rate loading. A diverse adoption of ML models leads to variations in prediction accuracy as well as the impact of input parameters on HPFRC fracture strength. Regarding the fine-tuning process described in Appendix A, it is important to note that further optimisation of hyperparameters, changes in ML algorithms and architectures, and the inclusion of additional input features may enhance the accuracy in predicting HPFRC fracture strength. The selection of machine learning models in the proposed framework, although intentionally diverse, remains flexible and may pave the way for future research endeavours, incorporating a hybrid evaluation system with continuous human feedback in the loop to develop a more sophisticated interactive prediction model.

Finally, it is important to acknowledge that the data collection used in this study relied on published data from established experiments with HPFRC at high strain rates [1,3,12,13,18]. Consequently, the findings of this study may be subject to the experimental designs employed in these sources. A future study with a more extensive collection of data may be needed to further validate the obtained findings. The establishment of computational simulation for HPFRC samples should be considered as a potential method for comparison with the results obtained from machine learning approaches. Further investigation on the application of the proposed framework on different types of mechanical properties (e.g., fracture energy, crack growth rate) is suggested for future research to develop a more comprehensive approach to characterise HPFRC structures.

## Additional information

No additional information is available for this paper.

## CRediT authorship contribution statement

**Quang Dang Nguyen:** Conceptualization, Data curation, Software. **Khoa Tan Nguyen:** Visualization, Writing – original draft, Writing – review & editing. **Tuan Kiet Tran:** Conceptualization, Investigation, Resources, Validation. **Kihak Lee:** Funding acquisition, Project administration, Supervision. **An Thao Huynh:** Formal analysis, Validation, Visualization, Writing – original draft, Writing – review & editing.

## Declaration of competing interest

The authors declare that they have no known competing financial interests or personal relationships that could have appeared to influence the work reported in this paper.

## Figures and Tables

**Table A.1:** Validation results of multiple deep learning models for predicting HPFRC fracture strength under the K-fold cross-validation scheme.

Config. Number	Network Architecture	Optimisation Method	Dropout	Normalisation Method	R^2^	RMSE	MAE
1	DNN	Adam	Yes	None	0.849	3.372	2.591
2	DNN	Adam	Yes	Batchnorm	0.848	3.388	2.586
3	DNN	Adam	Yes	Layernorm	0.886	3.059	2.221
4	Resnet	Adam	Yes	None	0.874	3.158	2.416
5	Resnet	Adam	Yes	Batchnorm	0.810	3.212	2.494
6	Resnet	Adam	Yes	Layernorm	0.886	2.956	2.297

**Table A.2:** Optimised hyperparameters of RF and XGBoost models for predicting HPFRC fracture strength using grid search, implemented with the scikit-learn library [45].

Method	Hyperparameters	
**Random Forest**	criterion = mse bootstrap = True, max features = auto	n_estimators = 1000 min_samples split = 2 min_samples leaf = 1,
**XGBoost**	objective = reg:squarederror n_estimators = 1000 nsubsample = 0.3, learning rate = 0.01,	max depth = 6, gamma = 0, colsample_bytree = 1.0,

**Fig. B.14:**
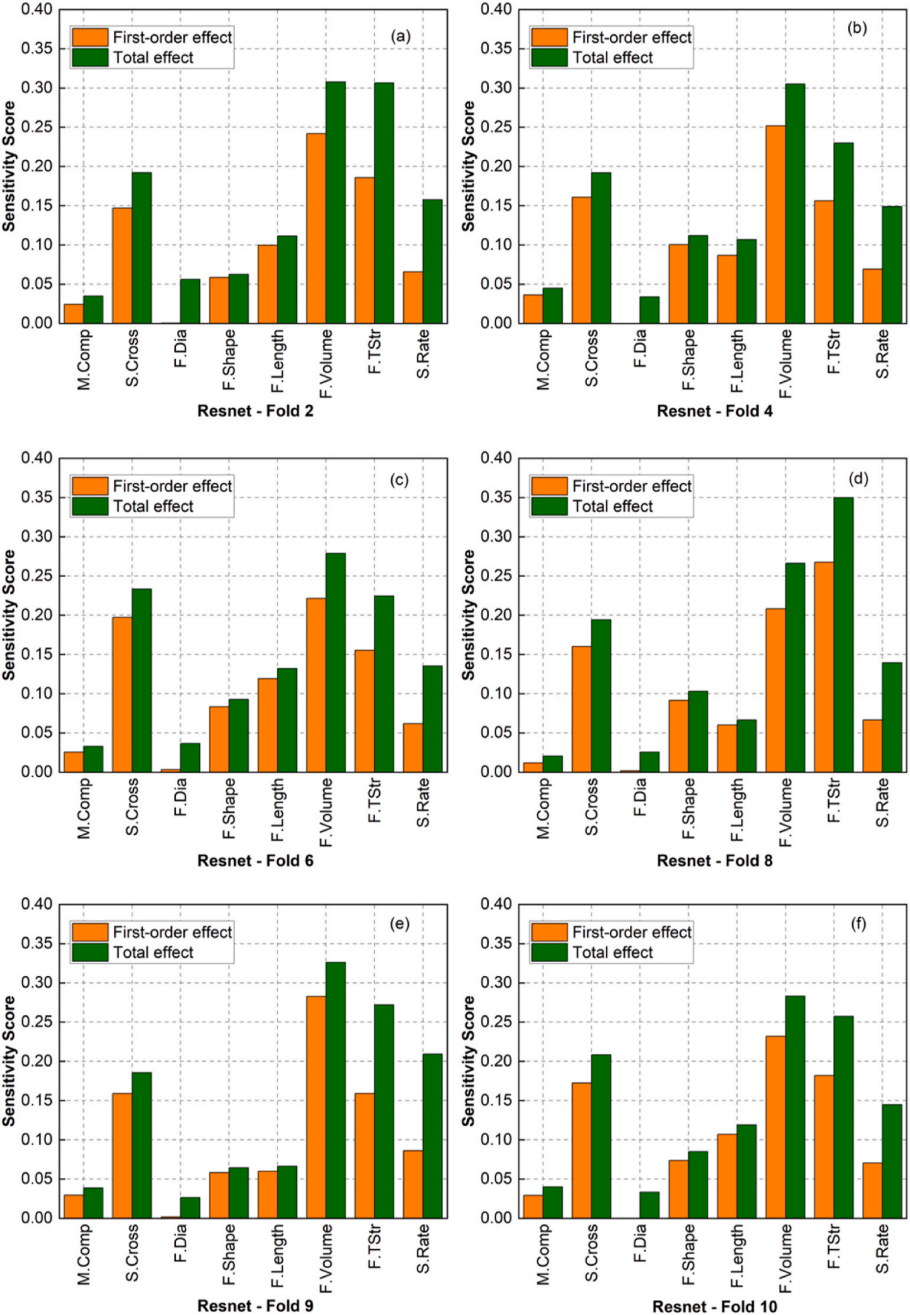
Sensitivity scores of first-order and total-order effects for ResNet models derived from multiple K-fold iterations in a K-fold cross-validation scheme..

## Appendix A Tuning hyperparameters for HPFRC fracture strength models

Four exemplary machine learning techniques were utilised to model the fracture strength of HPFRC subjected to high strain rates. Ensemble-based techniques, including RF and XGBoost, and feed-forward neural network solutions, such as DNN and ResNet, were employed. The hyperparameters for RF and XGBoost were optimised using grid search on popular ranges of key hyperparameters, while those for DNN and ResNet were determined based on the performance of six configurations listed in Table A1. A conventional two-hidden-layer ReLU-activated neural network with 256 and 128 nodes per layer was designed for the DNN model, while ResNet was constructed with an additional skip-connection weight layer containing 128 nodes (as shown in Fig. 4). The configurations with the Adam optimiser and LayerNorm normalisation appeared to be the optimal settings for DNN and ResNet, based on the average accuracy of the model's predictions under the K-fold cross-validation scheme (Table A1). The greatest accuracy among their respective types was achieved by RF and XGBoost models using the hyperparameters listed in Table A2. However, it should be noted that further optimisation of the hyperparameters and/or inclusion of additional input features may lead to even higher accuracy in predicting HPFRC fracture strength using the proposed machine learning models. Therefore, the framework presented in this study serves as a valuable starting point for future investigations aiming to improve the prediction performance and reliability of these models in material property analysis with limited datasets collected from small-scale experiments.
